# Decoction and Fermentation of Selected Medicinal Herbs Promote Hair Regrowth by Inducing Hair Follicle Growth in Conjunction with Wnts Signaling

**DOI:** 10.1155/2016/4541580

**Published:** 2016-03-24

**Authors:** Su Kil Jang, Seung Tae Kim, Do Ik Lee, Jun Sub Park, Bo Ram Jo, Jung Youl Park, Jong Heo, Seong Soo Joo

**Affiliations:** ^1^College of Life Science, Gangneung-Wonju National University, 120 Gangneung Daehangno, Gangneung, Gangwon 210-702, Republic of Korea; ^2^College of Pharmacy, Chung-Ang University, 221 Heukseok-dong, Dongjak-gu, Seoul 156-756, Republic of Korea; ^3^Industry-Academic Cooperation Foundation, Hanbat National University, Daejeon 305-719, Republic of Korea; ^4^Heo-jong Oriental Medicine Clinic, Mia-dong, Gangbuk-gu 62-3, Seoul 01205, Republic of Korea

## Abstract

It is well recognized that regulating the hair follicle cycle in association with Wnt signaling is one of the most interesting targets for promoting hair regrowth. In this study, we examined whether selected herbal medicines processed by decoction and fermentation promote hair growth by upregulating the number and size of hair follicles and Wnt signaling, including activation of *β*-catenin and Akt in telogen-synchronized C57BL/6N mice. The results revealed that the fermented extract after decoction (FDE) more effectively promoted hair growth than that of a nonfermented extract (DE). Notably, FDE effectively enhanced formation of hair follicles with clearer differentiation between the inner and outer root sheath, which is observed during the anagen phase. Mechanistic evidence was found for increased *β*-catenin and Akt phosphorylation levels in dorsal skin tissue along with elevated expression of hair regrowth-related genes, such as Wnt3/10a/10b, Lef1, and fibroblast growth factor 7. In conclusion, our findings suggest that FDE plays an important role in regulating the hair cycle by increasing expression of hair regrowth-related genes and activating downstream Wnt signaling targets.

## 1. Introduction

Hair is a defining property of mammals that plays important roles in keeping the body warm and dry and protecting against harmful environments. Thus, new hair is required constantly throughout life. In general, new hair is supplied by existing follicles through the anagen (growing phase of follicular epithelium), catagen (apoptosis and regression phase), and telogen phases (resting phase for the epithelium). A hair follicle is a skin organ that generates hair and has been the focus of stem cell studies [1, 2].

Hair follicles may be self-renewed by keratinocyte stem cells (KSCs) located at the bulge region, which contains undifferentiated cells [3, 4]. During the transition from the telogen to the anagen phases, Wnt signaling, which is required to establish the hair follicle and is upregulated only at the end of the telogen phase to promote entry into anagen, plays a key role in activating bulge stem cells to progress toward hair formation, and these signals are relayed in association with *β*-catenin and lymphoid enhancer factor 1 (Lef1) [5]. Wnt ligand expression is not detected in telogen phase follicles, and Wnt10a and 10b are only expressed at the onset of the anagen phase in the dermal papilla and secondary hair germ cells, respectively [6]. This is supported by the observation that *β*-catenin is generally confined to the membranes of bulge cells through most of the telogen phase and that nuclear *β*-catenin only becomes apparent in hair germ cells just before the follicle enters the anagen phase [5, 7].

To date, comprehensively effective highly safe candidate compounds prepared from herbal extracts have been examined for their hair regrowth activities. Among these, we selected eight herbs and derived an optimal extract through decoction and fermentation processes. In this study, we examined the hair regrowth activity of the extract in C57BL6/N mice and its prospective mechanism in conjunction with Wnt signaling.

## 2. Materials and Methods 

### 2.1. Preparation of the Study Sample

Eight selected medicinal herbs, such as* Cynanchum wilfordii, Mori Fructus, Schisandrae Fructus, Perillae Herba, Houttuyniae Herba, Ligustri Fructus, Longanae Arillus*, and* Polygonati Rhizoma*, were purchased from Saerom Pharmaceutical Co., Ltd. (Kyunggi, Republic of Korea). The study samples were prepared according to the method developed in our laboratory. In brief, to prepare the decoction, evenly weighed herbs (1 : 1) were finely ground and primarily immersed in autoclaved distilled water (1 : 10, w/v) for 30 min, followed by boiling on an electric heater for 2 h. The decoction was filtered using Whatman Grade No. 1 Filter Paper (Whatman International Ltd., Maidstone, UK) and centrifuged at 5000 rpm for 15 min. The supernatant was collected, lyophilized, and stored at 4°C before use. For fermentation,* Bacillus subtilis* was propagated twice in 50 mL MRS broth (Difco, Detroit, MI, USA) at 37°C overnight for fermentation. Then, 10^7^ CFU/mL of* B. subtilis* was inoculated into the decocted extracts and fermented at 37°C for 48 h (FDE). Nonfermented decoction extracts (DE) were used as a normal control. Both samples were serially filtered with a 60 *μ*m nylon net filter and a 0.22 *μ*m syringe filter (Millipore, Bedford, MA, USA), precipitated overnight, lyophilized (supernatant), and stored in desiccators at room temperature before use. During fermentation (24 and 48 h), samples of the liquid culture were examined under phase-contrast microscopy to visualize basic cell characteristics.

### 2.2. Animals

Healthy male C57BL6/N mice (7 weeks old) were obtained from Central Lab. Animal Inc. (Seoul, Republic of Korea) and were adapted to laboratory conditions (temperature: 20 ± 2°C, relative humidity: 50%, and light/dark cycle: 12 h) for 1 week. The animals (*n* = 3/group) were maintained at a constant temperature (23 ± 2°C), relative humidity (55 ± 10%), and a 12 h light/dark cycle and fed standard rodent chow and purified water* ad libitum*. Two days before the experiments, all mice (8 weeks of age) were shaved using animal clippers. Telogen-synchronized C57BL/6N mice were divided into six groups, each containing three male mice. The FDE and DE extracts as well as finasteride (FIN; Sigma-Aldrich, St. Louis, MO, USA) were administered daily for 20 consecutive days. The FDE and DE groups were given 64 or 128 mg/kg and 140 or 280 mg/kg, respectively, considering the vaporized solid parts of FDE (3.9 g/100 mL) and DE (8.7 g/mL). FIN (1 mg/kg) was administered as the positive at the same time points. All mice were sacrificed on day 21 and the dorsal hair growth patterns were photographed on days 0, 3, 6, 9, 12, 15, 18, and 20. The back area of each mouse was photographed with a digital camera and the image was inputted to a computer for measurement according to the following formula: [% Hair regrowth = hairy black area ÷ hair removal area] to quantitatively compare the hair regrowth patterns. The animal experiments were approved by the Gangneung-Wonju National University Animal Care and Use Committee (Approval number GWNU-2014-27), and all procedures were conducted in accordance with the Guide for Care and Use of Laboratory Animals published by the US National Institutes of Health.

### 2.3. Cell Culture

Human mesenchymal stem cells (hMSCs) used in this study were provided by Professor Kim (Chungbuk National University, Republic of Korea) [8, 9]. Cells were cultured at a density of 5 × 10^3^ cells/cm^2^ in complete medium and Dulbecco's modified Eagle's medium (DMEM, low glucose) was supplemented with 10% fetal bovine serum (FBS; Hyclone, Logan, UT, USA), 2.5 ng/mL hFGF2, 100 U/mL penicillin, and 100 *μ*g/mL streptomycin (Invitrogen, Carlsbad, CA, USA). Complete medium was changed every 2-3 days, and hMSCs were subcultured when they reach 1 × 10^4^ cells/cm^2^. Cultures were maintained under 5% CO_2_ at 37°C in tissue culture flasks.

### 2.4. Cellular Cytotoxicity (Lactate Dehydrogenase, LDH) Assay

The cytotoxicity induced by the FDE and DE was quantified by measuring LDH release. LDH content was determined using a commercial nonradioactive LDH assay kit, CytoTox 96® (Promega, Madison, WI, USA), which is based on a coupled enzymatic reaction that results in the conversion of a tetrazolium salt into a red formazan product. The increase in the amount of formazan produced in the culture supernatant directly correlates with the increase in the number of lysed cells. The formazan was quantified spectrophotometrically by measuring its absorbance at 490 nm (Spectra Max 340, Molecular Devices, Sunnyvale, CA, USA). Cytotoxicity in experimental samples was determined as % LDH release compared with that in cells treated with 1% Triton X-100.

### 2.5. Quantitative Real-Time Polymerase Chain Reaction (PCR) Assay

Total RNA from C57BL6/N dorsal skin tissues or hMSCs was prepared using the TRIZOL method (Invitrogen). cDNAs were synthesized from RNA by reverse transcription of 1 *μ*g total RNA using the ImProm-II reverse transcription system (Promega) and oligo dT primers in a total volume of 20 *μ*L. PCR amplification was performed using the primers described in Table 1 (Bioneer, Daejeon, Republic of Korea). Quantitative real-time PCR (qPCR) reactions were run on a Rotor-Gene 6000 (Corbett Research, Sydney, Australia) using SYBR Green PCR Master Mix (Qiagen, Valencia, CA, USA) in 20 *μ*L reaction mixtures. Each real-time PCR master mix contained 10 *μ*L 2x enzyme Mastermix, 7.0 *μ*L RNase free water, 1 *μ*L of each primer (10 pmole each), and 1 *μ*L diluted template. PCR was performed with an initial preincubation step for 10 min at 95°C, followed by 45 cycles of 95°C for 15 s, annealing at 52°C for 15 s, and extension at 72°C for 10 s. A melting curve analysis was used to confirm formation of the expected PCR product, and products from all assays were tested by 1.2% agarose gel electrophoresis to confirm the correct lengths. An interrun calibrator was used, and a standard curve was created for each gene to obtain PCR efficiencies. Relative sample expression levels were calculated using Rotor-Gene 6000 Series Software 1.7 and were expressed relative to glyceraldehyde 3-phosphate dehydrogenase and corrected for between-run variability. Data are expressed as a percentage of the internal control gene.

### 2.6. Western Blot Analysis

C57BL6/N dorsal skin tissues were homogenized and lysed in 1% RIPA buffer containing protease and phosphatase inhibitors (Roche, Mannheim, Germany), and total proteins were separated on 10% SDS-PAGE. After electrophoresis, the proteins were transferred to polyvinylidene fluoride membranes, and the membranes were blocked with 5% skim milk in Tris-buffered saline solution containing 0.1% Tween-20. The membranes were immunoblotted with primary antibodies, including anti-*β*-catenin, anti-phospo-Akt, anti-Akt, and anti-actin (Santa Cruz Biotechnology, Santa Cruz, CA, USA), followed by incubation with horseradish peroxidase-conjugated anti-rabbit or anti-mouse secondary antibodies (Stressgen, San Diego, CA, USA). The blots were developed using an enhanced chemiluminescent solution (Thermo, Rockford, IL, USA).

### 2.7. Histological Examination and Hair Follicle Count

Rectangular pieces of central dorsal skin were collected parallel to the vertebral line and fixed in 10% neutral buffered formalin (4 g sodium phosphate, monobasic, 6.5 g sodium phosphate, dibasic, 100 mL of 37% formalin, and 900 mL distilled water) for 5 days. The tissues were then embedded in paraffin, cut into sections (5 *µ*m), and stained with a hematoxylin-eosin (H&E) solution. The sections were deparaffinized with xylene, hydrated in a descending graded ethanol series, and stained with hematoxylin for 2 min, followed by 2 min washes and eosin staining for 5 s. All tissue samples were examined and imaged in a blinded fashion. Hair follicle counts were performed by using a digital photomicrograph and all of the images were cropped in a fixed area (300 pixels in width). Data were evaluated from representative areas at a fixed magnification of 100x. Images were captured using a Nikon Eclips Ti-S inverted microscope (Nikon, Tokyo, Japan) at 40x magnification.

### 2.8. Statistical Analysis

Statistical comparisons between groups were performed using one-way analysis of variance with Dunnett's post hoc test and SPSS v. 17 software (SPSS Inc., Chicago, IL, USA). A *P* < 0.05 was considered significant.

## 3. Results

### 3.1. Hair Growth-Promoting Action in Male C57BL6/N Mice

**Figure 1:**
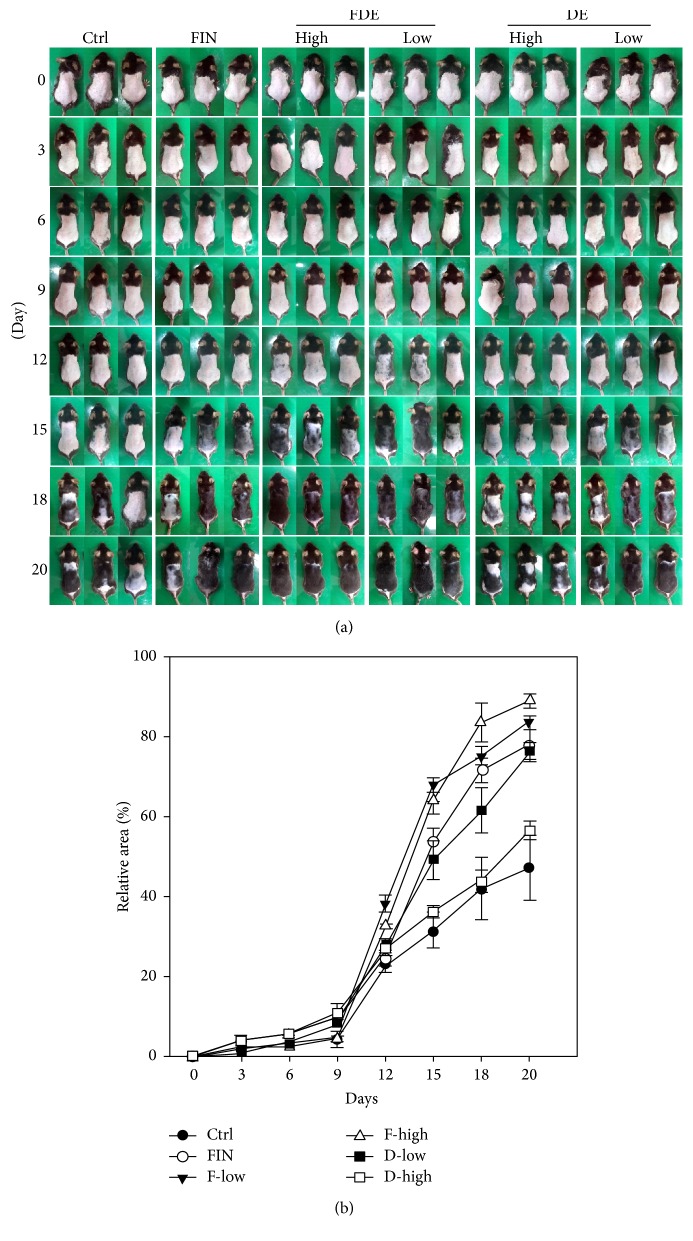
Hair growth-promoting effect in C57BL/6N mice. The dorsal skin of male C57BL/6N mice was shaved after the mice were orally administered the fermented herbal extract after decoction (FDE), the nonfermented herbal extract after decoction (DE), or finasteride for 20 days. (a) The shaved dorsal skin was photographed at 0, 3, 6, 9, 12, 15, 18, and 20 days. (b) The area of hair regrowth was measured by image software on the indicated day. FIN, finasteride; F, FDE; D, DE.

Hair growth patterns in the shaved area were compared in all groups. To evaluate the hair growth effect, FIN (1 mg/kg), FDE (low: 64 mg/kg, high: 128 mg/kg), and DE (low: 140 mg/kg, high: 280 mg/kg) were administered, and patterns of dorsal hair growth were examined on days 0, 3, 6, 9, 12, 15, 18, and 20 (Figure 1(a)). As shown in Figure 1(a), the FDE and DE caused a gray hair color on day 12 after induction, and the hair shafts were visible on day 15, whereas hair in the control group remained unpigmented until day 15. Interestingly, regrowth of hair in FDE-treated mice was as much as that seen in the FIN positive control, which is a representative oral hair loss drug used worldwide (Figure 1(b)).

### 3.2. Effects of FDE/DE on Hair Follicle Structure

**Figure 2:**
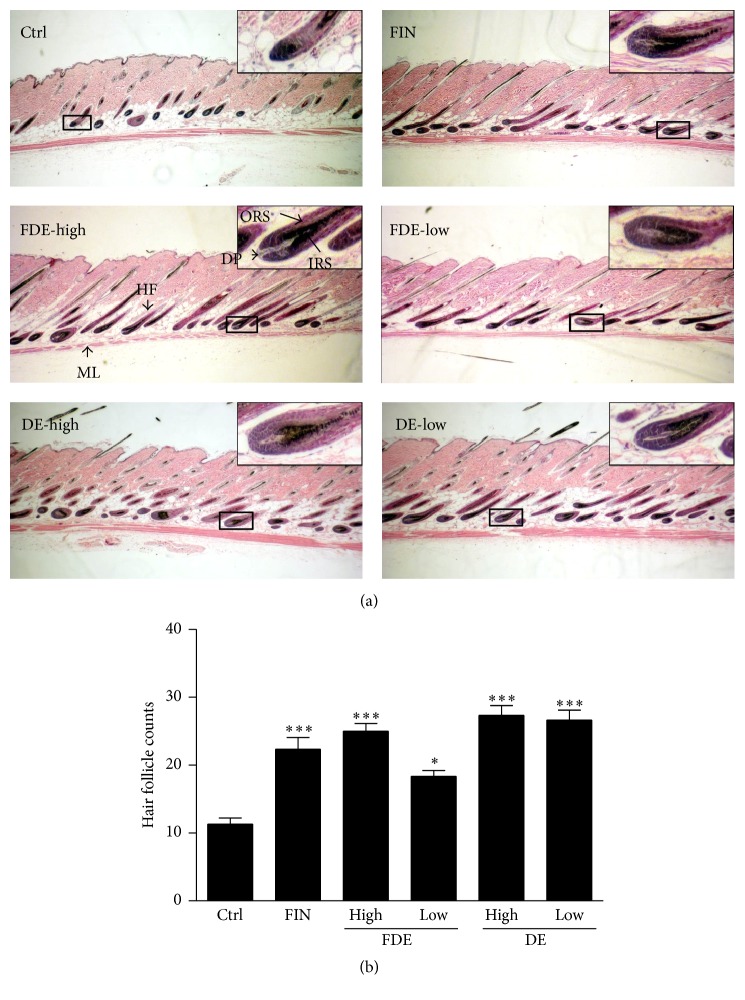
Comparison of hair follicle growth in C57BL/6N mice. (a) Hematoxylin-eosin staining of dorsal skin from mice administered the fermented herbal extract after decoction (FDE), the nonfermented herbal extract after decoction (DE), or finasteride for 20 days was analyzed. Arrows are muscle layer (ML), dermal papilla (DP), outer root sheath (ORS), and inner root sheath (IRS). (b) The number of hair follicles in deep subcutis. Values are mean ± standard deviations. ^*∗*^ *P* < 0.05, ^*∗∗∗*^ *P* < 0.001 versus Ctrl. Ctrl, control group.

Hair follicle growth (anagen) between groups was compared in accordance with accepted morphological guidelines [10]. Our results revealed that FDE and DE increased the number and size of hair follicles, which are markers for transition of follicles from the telogen to anagen phase of hair growth, whereas hair in the control group was in the early anagen phase, in which enlarged dermal papilla and the bulb located in the dermis are distinct characteristics (Figure 2(a)). Notably, H&E staining of FDE and DE mice well demonstrated that the hair follicles were at least in the anagen IIIc-IV phase, representing thinner dermal papilla, maximal hair bulb size and volume, and newly formed hair shafts. The number of hair follicles in the longitudinal sections of the FDE-treated mice increased similar to that observed in the positive FIN control (Figure 2(b)).

### 3.3. Expression of Wnt, Fibroblast Growth Factor (FGF) 7, and Lef1 Genes in C57BL6/N Dorsal Skin

**Figure 3:**
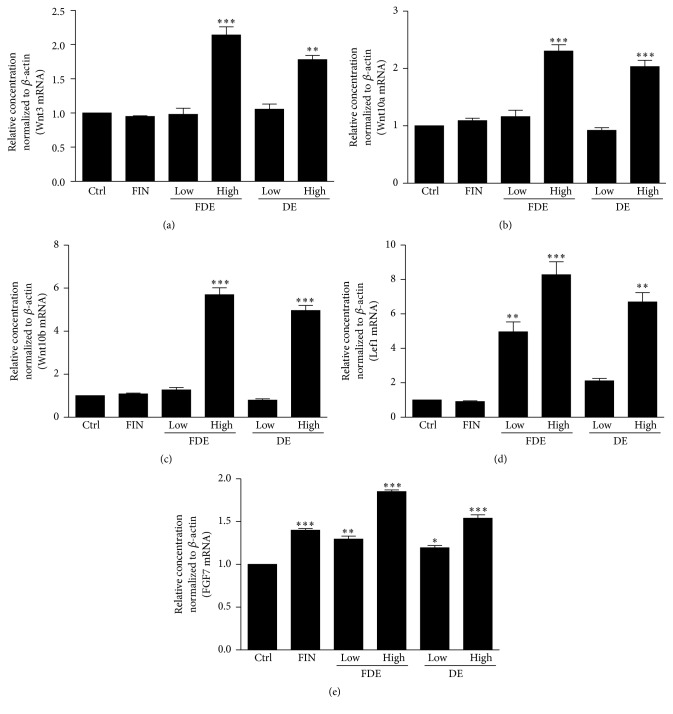
Comparative analysis of the expression of hair regrowth-related genes at the mRNA level in dorsal skin tissue. The tissues were collected on day 21, and mRNAs were harvested using TRIZOL. Wnt3/10a/10b, Lef1, and fibroblast growth factor (FGF) 7 genes were compared by real-time quantitative polymerase chain reaction. Results are expressed as means ± standard deviations. ^*∗*^ *P* < 0.05, ^*∗∗*^ *P* < 0.01, and ^*∗∗∗*^ *P* < 0.001 versus Ctrl. Ctrl, control group.

Primary Wnt (Wnt3) and secondary Wnts (Wnt10a and Wnt10b) are essential for hair follicle initiation, morphogenesis, and development [11] and were examined to determine whether FDE and DE increased expression of dermal Wnt genes implicated in the first signal essential for inducing hair follicles. Figures 3(a)–3(c) indicate that oral administration of FDE and DE contributed largely to differentiation of the hair medulla and regulation of matrix/precortex cells by stimulating Wnt genes (3, 10a, and 10b) at the higher concentration. Moreover, FDE and DE upregulated expression of Lef1, which is an essential regulatory gene in the Wnt signaling pathway that controls cell growth and differentiation through a signaling cascade from Wnts to Lef1 (Figure 3(d)). The FGF7 gene was also overexpressed in the FDE- and DE-treated groups (Figure 3(e)), suggesting a prolonged anagen phase and delayed progression into the catagen phase in dermal papilla cells [12].

### 3.4. Activation of *β*-Catenin and Akt Signaling by FDE and DE

**Figure 4:**
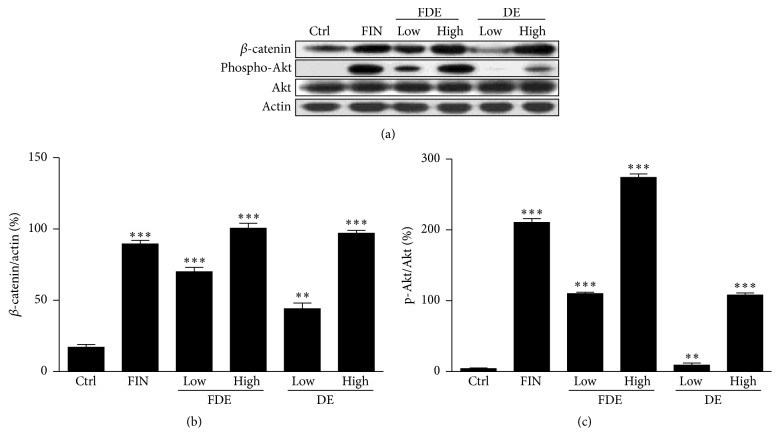
Western blot analysis of *β*-catenin and Akt phosphorylation. (a) Immunoblotting analysis of *β*-catenin and phospho-Akt protein levels was conducted in C57BL6/N dorsal skin tissue sampled on day 21 after treatment. (b) A bar graph shows the quantification of *β*-catenin/actin and (c) phospho-Akt/Akt ratio. Finasteride (FIN) was used as the positive control. ^*∗∗*^ *P* < 0.01, ^*∗∗∗*^ *P* < 0.001 versus Ctrl. Ctrl, control group.

Cytosolic *β*-catenin, an essential molecule in the Wnt signaling pathway, translocates into the nucleus where it induces transcription of target genes. Thus, we investigated whether FDE or DE upregulates *β*-catenin levels in dorsal skin tissues. *β*-catenin level increases during the initial stages of hair regeneration. We also compared the protein level of phosphorylated Akt, a downstream target of phosphoinositide 3-kinase, with that in the FIN group. *β*-catenin expression increased in response to the high doses of FDE and DE to levels comparable to those in the FIN group. Moreover, Akt activation, which promotes hair regrowth by regulating dermal papilla cell proliferation in the hair follicle, was upregulated in the presence of FDE and DE, with more upregulation in the FDE than that in the DE (Figures 4(a)–4(c)).

### 3.5. Cytotoxicity and Profiles of Wnts mRNAs in hMSCs

**Figure 5:**
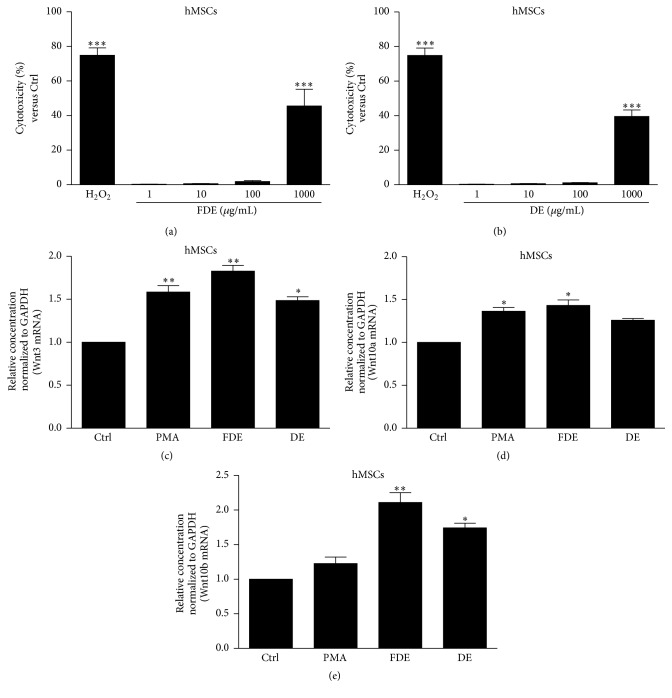
Effect on Wnts (Wnt3, 10a, and 10b) gene expression in hMSCs. (a-b) Cytotoxicity assays were performed in a 96-well plate for 24 h, and the results were expressed as the percent cytotoxicity for identical treatments of HDE and DE (1–1000 *μ*g/mL). For gene expression analysis, cells were seeded on a 6-well plate and treated with the HDE (100 *μ*g/mL) or DE (100 *μ*g/mL) in the presence or absence of 50 *μ*M phorbol myristic acetate (PMA) for 24 h. (c–e) Wnt3, Wnt10a, and Wnt10b mRNA were quantified by fold units using the real-time polymerase chain reaction. Results are expressed as means ± standard deviations from three separate experiments. ^*∗*^ *P* < 0.05, ^*∗∗*^ *P* < 0.01, and ^*∗∗∗*^ *P* < 0.001 versus Ctrl. Ctrl, control group.

The cytotoxicity induced by the FDE and DE was quantified by measuring LDH release at varying ranges of concentration (1–1000 *μ*g/mL). Incubating hMSCs with each of the study samples did not result in cell cytotoxicity except at the highest dose (1000 *μ*g/mL). Figures 5(a) and 5(b) show that the FDE and DE did not significantly increase LDH release for 1–100 *μ*g/mL exposure for up to 24 h but a higher concentration (1000 *μ*g/mL) induced a significant increase in LDH release. Figures 5(c)–5(e) clearly show that FDE significantly increased Wnt3, Wnt10a, and Wnt10b mRNA in hMSCs. As expected, results of this* in vitro* study coincide with the* in vivo* dorsal skin results.

## 4. Discussion

The hair follicle is a mammalian skin organ that produces hair, which is repeatedly renewed through canonical signaling, for proper growth and patterning. Otherwise, the hair cycle is disrupted, and hair is lost from the head or body, which is called alopecia. To date, two representative types of drugs have been prescribed for pattern baldness. FIN, which is a 5 alpha-reductase inhibitor, and minoxidil, which is an antihypertensive vasodilator, are the most frequently prescribed medications for alopecia. The selected herbal medicines used in this study have been reported to have diverse pharmacological activities, such as antihypercholesterolemia, immunoregulation, antiallergy, antibacteria, antioxidation, anticancer, hepatoprotection, and anti-insomnia [13–18].

As C57BL/6N mice have been shown to be in a synchronized telogen stage of the hair cycle at 8 weeks of age, their hair follicle regeneration and regrowth have been well described. The high dose of FDE promoted hair regrowth during the 20 consecutive days of administration that was equal to that seen in the positive FIN control. Wnt signaling is required to establish the hair follicle [19] and plays a key role activating bulge stem cells to progress toward hair formation, and this signal is relayed by *β*-catenin and Lef1 [5].

In recent studies, a hierarchy of Wnts that control hair follicle development has been introduced. According to the prior reports, among 19 Wnts, Wnts 3, 4, and 6 were considered to mediate hair follicle initiation and Wnts 2, 7b, 10a, and 10b were considered to depend on the epidermal activation [6, 10, 20]. In line with this, Wnt secretion mediates hair follicle development by acting as signaling molecules and they can be classified as primary Wnts (Wnts 3, 4, and 6) and secondary Wnts (Wnts 2, 7b, 10a, and 10b), which are essential for initiating hair follicle growth and are involved in development of the hair follicle, respectively [10, 21]. In particular, matrix and precortex cells express Wnt3 and Wnt10a/b, which send a signaling cascade to Lef1 [22, 23]. Importantly, *β*-catenin, which is a key regulator of hair follicle growth, is involved in inducing the transition from telogen to anagen [23]. Thus, it is important that a hair regrowth candidate should be associated with Wnt signaling. H&E staining demonstrated that FDE and DE actively promoted hair follicle regrowth by increasing the number and size of hair follicles, which is an indicator for the transition of hair growth from the telogen to anagen phases at particular times. The hair of FDE-treated mice was more prone to transition into the late anagen phase (i.e., IIIc), in which the dermal papilla reaches the deepest position and rests close to the muscle layer along with the distinguishable inner and outer root sheaths [10].

The increase in Wnt3/10a/10b and Lef1 gene expression in the dorsal skin strongly indicates that hair follicles were actively developing because these genes are predominantly expressed in the hair placode epithelium during induction of the hair follicle [24–26]. Coincident data were obtained from qPCR analysis in hMSCs, which are known to express all of the common stem cell markers and can be differentiated into various types of specialized cells under appropriate growth conditions [27]. Taken together, FDE could play an essential role in the initiation and development of hair follicles, as shown by the H&E staining results. Furthermore, the increase in FGF7 mRNA helps understand how FDE prolongs the anagen phase and delays progression into the catagen phase.

## 5. Conclusion

Our data clearly demonstrate that FDE effectively promotes hair regrowth by enhancing Wnt signaling and activating Akt together with overexpression of hair regrowth-related primary and secondary Wnts (Wnt3/10a/10b), Lef1, and FGF7. Although more in-depth chemical screening studies are required, it is certain that the extract from eight selected herbal medicines could be a promising hair regrowth candidate when fermented after decoction.

## Figures and Tables

**Table 1:** Primer sequences used for the real-time polymerase chain reaction analysis.

Gene		Primer	Amino acid sequences	Product size (bp)	Accession number
Mouse	FGF7	5′ primer	5′-TGCTTCCACCTCGTCTGTCT	212	NM_008008
		3′ primer	5′-GAGGCAAAGTGAAAGGGACC		
	Wnt3	5′ primer	5′-AGAGACGGGCTCCTTTGGTA	123	NM_009521
		3′ primer	5′-TTCTCCTTCCGTTTCTCCGT		
	Wnt10a	5′ primer	5′-GTGCGCTCTGGGTAAACTGA	232	NM_009518
		3′ primer	5′-AGAGAAGCGTTCTCCGAAGC		
	Wnt10b	5′ primer	5′-TCTTGGCTTTGTTCAGTCGG	124	NM_011718
		3′ primer	5′-CCCAGCTGTCGCTTACTCAG		
	*β*-catenin	5′ primer	5′-AGGCTTTTCCCAGTCCTTCA	122	M90364
		3′ primer	5′-TCTGCATGCCCTCATCTAGC		
	Lef1	5′ primer	5′-CGTCCTCTCAGGAGCCCTAC	169	X58636
		3′ primer	5′-GGAGAAAGGGACCCATTTGA		
	*β*-actin	5′ primer	5′-TACAGCTTCACCACCACAGC	187	NM_007393
		3′ primer	5′-AAGGAAGGCTGGAAAAGAGC		

Human	Wnt3	5′ primer	5′-CACATGCACCTCAAATGCAA	132	AB067628
		3′ primer	5′-CGAGGCGCTGTCATACTTGT		
	Wnt10a	5′ primer	5′-TTCCACTGGTGCTGCGTAGT	107	AB059570
		3′ primer	5′-CTGCGCGAAGTCAGTCTAGC		
	Wnt10b	5′ primer	5′-CATACAGGGCATCCAGATCG	148	AB059569
		3′ primer	5′-AAAAGCGCTCTCTCGGAAAC		
	GAPDH	5′ primer	5′-GGAGCCAAAAGGGTCATCAT	203	AK_026525
		3′ primer	5′-GTGATGGCATGGACTGTGGT		
